# Therapeutic Evaluation of Synthetic Peucedanocoumarin III in an Animal Model of Parkinson’s Disease

**DOI:** 10.3390/ijms20215481

**Published:** 2019-11-04

**Authors:** Sangwoo Ham, Heejeong Kim, Jin-Ha Yoon, Hyojung Kim, Bo Reum Song, Jeong-Yun Choi, Yun-Song Lee, Seung-Mann Paek, Han-Joo Maeng, Yunjong Lee

**Affiliations:** 1 Division of Pharmacology, Department of Molecular Cell Biology, Sungkyunkwan University School of Medicine, Samsung Biomedical Research Institute, Suwon 16419, Korea; ham89p12@skku.edu (S.H.); kimhj9301@gmail.com (H.K.); hjung93@skku.edu (H.K.); choijy@skku.edu (J.-Y.C.); yslee@skku.edu (Y.-S.L.); 2 College of Pharmacy, Gachon University, Incheon 21936, Korea; jinha89@daum.net; 3 College of Pharmacy and Research Institute of Pharmaceutical Sciences, Gyeongsang National University, Jinju Daero 501, Jinju 52828, Gyeongnam, Korea; qhfma2005@naver.com

**Keywords:** peucedanocoumarin III, organic synthesis, Parkinson’s disease, distribution, α-synuclein aggregation, dopaminergic cell loss

## Abstract

The motor and nonmotor symptoms of Parkinson’s disease (PD) correlate with the formation and propagation of aberrant α-synuclein aggregation. This protein accumulation is a pathological hallmark of the disease. Our group recently showed that peucedanocoumarin III (PCIII) possesses the ability to disaggregate β sheet aggregate structures, including α-synuclein fibrils. This finding suggests that PCIII could be a therapeutic lead compound in PD treatment. However, the translational value of PCIII and its safety information have never been explored in relevant animal models of PD. Therefore, we first designed and validated a sequence of chemical reactions for the large scale organic synthesis of pure PCIII in a racemic mixture. The synthetic PCIII racemate facilitated clearance of repeated β sheet aggregate (β23), and prevented β23-induced cell toxicity to a similar extent to that of purified PCIII. Given these properties, the synthetic PCIII’s neuroprotective function was assessed in 6-hydroxydopamine (6-OHDA)-induced PD mouse models. The PCIII treatment (1 mg/kg/day) in a 6-OHDA-induced PD mouse model markedly suppressed Lewy-like inclusions and prevented dopaminergic neuron loss. To evaluate the safety profiles of PCIII, high dose PCIII (10 mg/kg/day) was administered intraperitoneally to two-month-old mice. Following 7 days of PCIII treatment, PCIII distributed to various tissues, with substantial penetration into brains. The mice that were treated with high dose PCIII had no structural abnormalities in the major organs or neuroinflammation. In addition, high dose PCIII (10 mg/kg/day) in mice had no adverse impact on motor function. These findings suggest that PCIII has a relatively high therapeutic index. Given the favorable safety features of PCIII and neuroprotective function in the PD mouse model, it may become a promising disease-modifying therapy in PD to regulate pathogenic α-synuclein aggregation.

## 1. Introduction

Parkinson’s disease (PD) is a progressive neurodegenerative movement disorder of which characteristic pathological features include Lewy bodies, intracellular inclusions that are mainly composed of misfolded and phosphorylated α-synuclein [1]. Pathological Lewy bodies in PD are first found in the lower brain stem, after which they spread to the midbrain and finally to the cortex in the advanced stages [2,3,4]. This pathological a-synuclein aggregation in diverse brain subregions corresponds to the progression of clinical motor and nonmotor symptoms in PD [2,3,4]. Currently available treatments for the motor and non-motor symptoms of PD address only symptoms. In particular, L-DOPA supplements increase dopamine neurotransmission, and therefore control the clinical motor deficits in PD. However, the therapeutic effects of L-DOPA decline as the disease progresses. This treatment eventually results in adverse effects such as dyskinesia. Therefore, it is imperative to develop disease-modifying therapies that halt or reverse the ongoing pathological propagation of α-synuclein pathologies and neurodegeneration. Specifically, therapeutic strategies to regulate Lewy body inclusion may hold promise in controlling pathological α-synuclein propagation in various brains subregions. These strategies may have more therapeutic value in treating the motor and nonmotor symptoms that are caused by Lewy body pathology.

Several compounds have been reported to have disaggregating ability against fibrillar α-synuclein in in vitro or cell systems. However, only a few compounds have been evaluated in animal models of PD. For instance, Nilotinib, the brain permeable c-Abl inhibitor, has been shown to reduce **α**-synuclein aggregation and dopaminergic neurodegeneration in PD mouse models [5,6]. In addition, the stable GLP-1 agonist was also shown to control **α**-synuclein pathologies via regulation of microglia and astrocyte conversion [7]. In addition to these therapeutic evaluations in PD mouse models, extensive safety profiling of potential lead compounds are also important. This is particularly true for translational research aiming for clinical trials in the future. In order to control for the potential toxicity of prolonged therapies in neurodegenerative disorders, it is essential to understand the potential alterations in organ integrities or behavior in mice with the treatment.

Our group previously identified that peucedanocoumarin III (PCIII) has strong inhibitory properties against artificially repeated β sheet aggregate toxicity [8]. In addition, PCIII disaggregated α-synuclein fibrils in vitro and prevented **α**-synuclein induced toxicity in cells. In this case, PCIII purified from A. decursiva roots [8] was used. Despite PCIII’s therapeutic potential for PD, translational research has not been attempted because of issues with stable-and large-scale production of qualitatively consistent PCIII. In this study, we developed an organic synthesis protocol for highly pure PCIII production. We demonstrated therapeutic functions of PCIII in the regulation of α-synuclein aggregation and dopamine cell viability in a PD mouse model. Importantly, we also thoroughly evaluated the tissue distribution and safety information of synthetic PCIII in vivo.

## 2. Results

### 2.1. Organic Synthesis of Peucedanocoumarin III and its Evaluation

**Figure 1:**
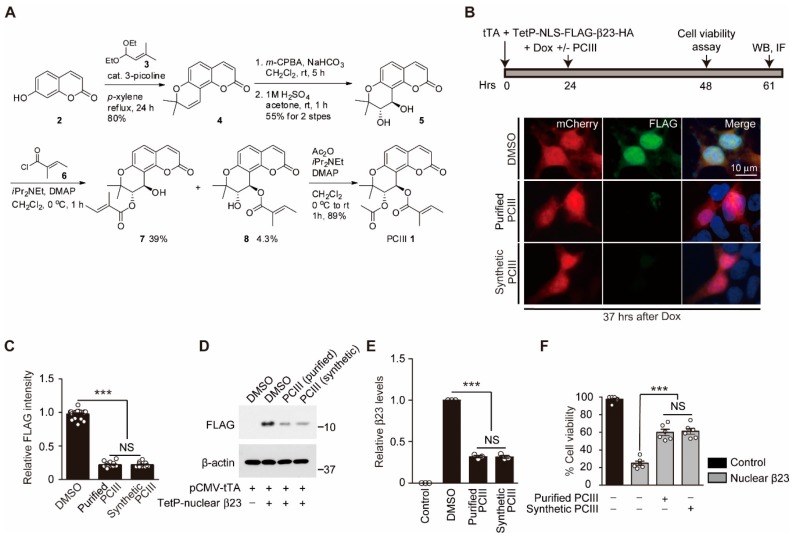
Total synthesis of peucedanocoumarin III (PCIII) and evaluation of its anti-aggregate effect. (**A**) Scheme showing the detailed experimental steps in the organic synthesis of PCIII. (**B**) Experimental schedule used to evaluate synthetic PCIII’s protective effect on Tet-Off-expressed β23 toxicity (top panel). Nuclear β23 was expressed for 24 h using the Tet-off system. Further expression of β23 was halted by doxycycline (Dox) treatment. Cell viability was assessed 24 h after doxycycline treatment. The degradation of β23 was monitored by Western blot (WB) and immunofluorescence (IF) at 37 h after doxycycline treatment. The representative immunofluorescence images of FLAG (β23) and the reporter protein mCherry in SH-SY5Y cells treated with the natural compound PCIII, synthetic PCIII (1 µM, 37 h), or DMSO as a vehicle. (**C**) Quantification of relative nuclear FLAG (β23) immunofluorescence intensities in the reporter mCherry positive SH-SY5Y cells from the indicated experimental groups (*n* = 21 cells from three independent experiments per groups). (**D**) Representative Western blot of nuclear β23 expression in SH-SY5Y cells treated with either natural or synthetic PCIII or DMSO vehicle. β23 degradation was monitored using anti-FLAG antibodies. The control included the SH-SY5Y cells transfected with pCMV-tTA and TetP mock vector without β23 expression. β-actin served as a loading control. (**E**) Quantification of relative nuclear β23 protein levels normalized to β-actin for the indicated experimental groups (*n* = 3 per group). (**F**) Cell viability assessed using trypan blue exclusion assay (*n* = 6 per group), which showed the protective effect of both purified and synthesized PCIII (1 µM each) in nuclear β23-expressing (48 h) SH-SY5Y cells. The control included the SH-SY5Y cells, which were transfected with pCMV-tTA and TetP mock vector without β23 expression. The data are expressed as means ± SEMs. *** *p* < 0.001, ANOVA test followed by Tukey post hoc analysis. NS, nonsignificant.

To ensure consistent and reliable therapeutic outcomes with in vivo PCIII treatment, it is essential to standardize the large scale organic synthesis of highly pure PCIII. We designed chemical reaction procedures for the organic synthesis of PCIII. The detailed chemical synthesis of PCIII **1** is outlined in Figure 1A. Umbelliferone **2** was treated with acetal 3 followed by cyclization under thermal conditions to produce good yield of seselin [9,10,11]. *m*CPBA epoxidation of alkene in seselin **4** afforded corresponding racemic epoxide, which was transformed into trans-diol **5** through acidic hydrolysis [12,13]. We then tried selective esterification of the trans-diol moiety. Some acylating agents such as tigloyl chloride or acetic anhydride were esterified under weak basic conditions to provide the desired intermediate **8** or its synthetic equivalents. The differentiation of two secondary alcohols, however, was not productive after survey of the reaction conditions. Finally, the desired ester **8** was obtained as a minor product of tigloylation. The undesired isomer **7** [14] was a major product. The standard acetylation protocol then afforded PCIII as a racemate, which is identical to the authentic spectral data [8,14]. The PCIII was produced in excellent yield. It was interesting that the acetylation/tigloylation sequence did not produce PCIII because of its weak reactivity.

Since synthetic PCIII is produced as a racemate, we attempted to compare the biological functions of synthetic PCIII with purified PCIII (that is extracted from natural herbs, as described previously) [8]. To determine whether synthetic PCIII retains the ability to degrade artificial beta sheet structured protein aggregate β23 [8,15], the SH-SY5Y cells were transiently transfected with both pCMV-tTA and TetP-NLS-FLAG-β23-HA (TetP-β23) to produce doxycylcline-regulatable expression of aggregate β23 (Figure 1B, top panel). The steady state levels of β23 were monitored in the SH-SY5Y cells expressing reporter mCherry using immunofluorescence. This immunofluorescence was performed by labeling the N terminal tag FLAG with the specific antibodies. In comparison to purified PCIII, the synthetic PCIII treatment accelerated β23 clearance from the SH-SY5Y cells to a comparable extent (Figure 1B,C). We next sought to further confirm that the synthetic PCIII treatment degraded β23. To do so we exposed total protein lysates from the SH-SY5Y cells (pulsed 24 h expression of β23) to Western blots using FLAG specific antibodies. The sustained presence of β23 in the DMSO vehicle group was cleared up to approximately 60% with the purified and synthetic PCIII treatment (Figure 1D,E). Synthetic PCIII provided potent cytoprotection against β23 in a dose-dependent manner, with maximal protective effect at 1 μM concentration (Supplementary Figure S1A). In addition, consistent with the degradation of β23, β23-induced ~80% cell death in DMSO control group was reduced to around 40% cell death after treatment of either purified or synthetic PCIII (Figure 1F). We also examined PCIII’s cytoprotective function in another PD-related cellular model, 6-OHDA-intoxicated SH-SY5Y cell that has been reported to produce α-synuclein aggregation and cytotoxicity [8,16]. Following HA-α-synuclein transient transfection to SH-SY5Y cells, subsequent 6-OHDA treatment led to robust cell toxicity of about 80% cell death (Supplementary Figure S1B). Synthetic PCIII treatment provided partial cytoprotection in this PD-related cell model (Supplementary Figure S1B). These results suggest that synthetic PCIII has similar biologic activity to that of purified PCIII, including β23 degradation and cytoprotection against β23 and 6-OHDA-induced toxicity.

### 2.2. PCIII Prevents DA Cell Loss and α-synuclein Aggregation in a 6-OHDA PD Mouse Model

**Figure 2:**
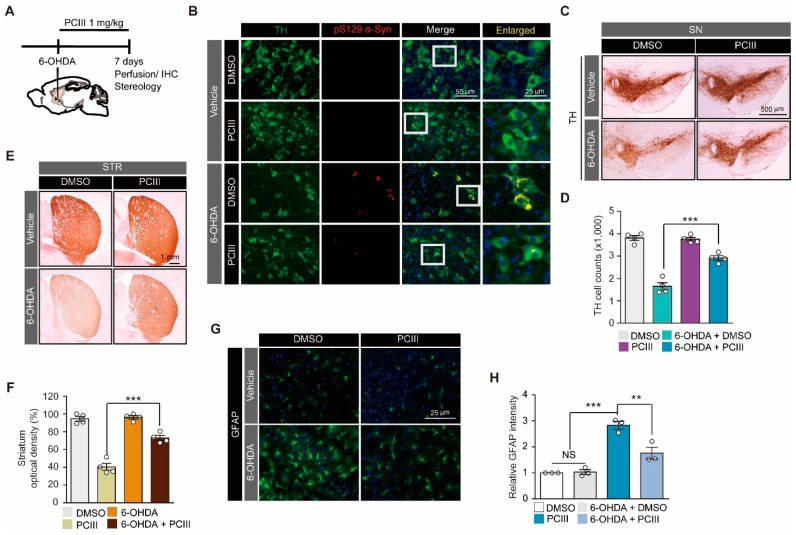
PCIII prevents 6-OHDA-induced α-synuclein aggregation and dopamine cell death in vivo. (**A**) The schematic experimental schedule of PCIII therapeutic evaluation in 6-OHDA (8 µg) induced PD mouse models. IHC, immunohistochemistry. To model the dopaminergic neuronal death in mice, 6-OHDA was stereotaxically injected into the striatum (coordinates from bregma, L: -2.0, AP: 0.5, DV: -3.0 mm) of 2-month-old mouse brains. (**B**) Representative immunofluorescence images of TH (green) and serine 129-phosphorylated α-synuclein (pS129 α-Syn, red) expression in the ventral midbrain sections from 6-OHDA-induced PD mice treated with PCIII (i.p. 1mg/kg/day) or DMSO for seven days. (**C**) Representative tyrosine hydroxylase (TH) immunohistochemical staining of the substantia nigra of the 6-OHDA PD mice treated with PCIII (i.p. 1 mg/kg/day, 7 days) or DMSO. (**D**) Stereological assessment of tyrosine hydroxylase (TH)-positive dopaminergic neurons in the substantia nigra pars compacta of the injection side of the indicated mouse groups (*n* = 4 mice per group). (**E**) Representative tyrosine hydroxylase (TH) immunohistochemical staining of the striatum (STR) of 6-OHDA PD mice treated with PCIII (i.p. 1 mg/kg/day, 7 days) or DMSO. (F) Quantification of relative TH stained optical dopaminergic nerve terminal fiber densities in the striatum of the indicated experimental groups (*n* = 4 mice per group). (**G**) Representative immunofluorescence images of GFAP in the substantia nigra of the indicated mouse groups. (**H**) Quantification of relative GFAP fluorescence intensities in the substantia nigra sections from the indicated experimental groups (*n* = 3 mice per group). The quantified data are expressed as means ± SEMs. Statistical significance was determined by the ANOVA test with Tukey post-hoc analysis, ** *p* < 0.01, and *** *p* < 0.001. NS, nonsignificant.

We previously reported that PCIII treatment can prevent oxidative stress-induced α-synuclein aggregation and toxicity in cell systems [8]. In order to reproduce this observation in vivo, we used an intrastriatal injeciton of 6-OHDA as a mouse model of PD (Figure 2A). The 6-OHDA-induced PD mouse model has been widely used to model dopaminergic neuronal degeneration [17,18] and α-synuclein aggregation [16]. To evaluate the protective function of synthetic PCIII, PCIII was administered intraperitoneally (1mg PCIII per kg mouse body weight) daily for 7 days following 6-OHDA intrastriatal stereotaxic injection (Figure 2A). Consistent with the previous report [16,19], 6-OHDA intoxication produced a phospho-serine 129 α-synuclein (pS129-α-Syn) positive Lewy like inclusion in the TH-labeled dopaminergic neurons (Figure 2B). The PCIII treatment substantially reduced pS129 α-synuclein intensities (Figure 2B), which indicate prevention or degradation of 6-OHDA-induced Lewy like inclusion formation.

The dopaminergic neuronal viability was assessed using immunohistochemistry with anti-tyrosine hydroxylase (TH) specific antibodies. The TH-stained dopamine neurons in the substantia nigra pars compacta were counted via unbiased stereological counting. The PBS-injected side of the ventral midbrains showed approximately 4000 dopaminergic cells after i.p. administrations of either DMSO or PCIII (Figure 2C,D). This result indicates that the PCIII treatment did not have overt dopaminergic toxicity. On the other hand, PCIII treatment in 6-OHDA PD mouse models largely alleviated the 6-OHDA-induced dopamine cell loss (Figure 2C,D). The loss of striatal dopaminergic nerve terminals because of 6-OHDA intoxication in vivo was also substantially protected by PCIII treatment, as demonstrated by TH optical density measurements in the striatum (Figure 2E,F). We further evaluated the potential neuroinflammation using GFAP immunofluorescence, which labels astrogliosis. The 6-OHDA injection increased GFAP immunofluorescence signal in the ventral midbrain approximately three-fold higher than did the PBS injection in controls (Figure 2G,H). The PCIII treatment reduced the GFAP signal (that was increased by 6-OHDA) by about two-fold compared to that of the PBS injection control (Figure 2G,H). Taken together, these results demonstrated that synthetic PCIII has neuroprotective functions in the 6-OHDA PD mouse model. The PCIII treatment also prevented Lewy body-like structures of pS129-α-synuclein in dopamine neurons of the PD mouse model. This result reflects the therapeutic potential of PCIII in PD treatment.

### 2.3. Safety Profiling of Synthetic PCIII Administration In Vivo

**Figure 3:**
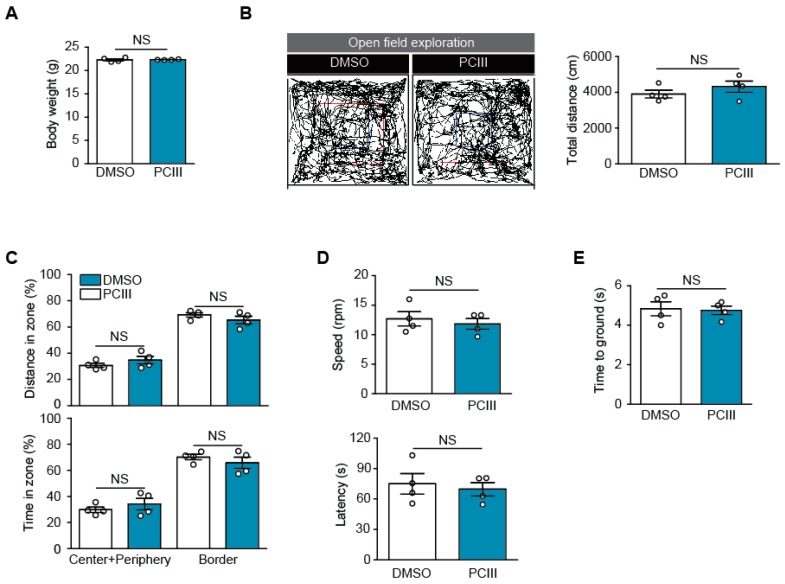
Safety profiling of PCIII in vivo. (**A**) The body weights of mice treated with high dose PCIII (i.p., 10 mg/kg/day) or DMSO vehicle for 7 days. (**B**) The spontaneous exploration of mice treated with high dose PCIII or DMSO traced by the open field test (left panel). The blue box is a center area, and the red box is a periphery area. The area inside of the red box is “Center+Periphery” zone, and the area outside of the red box is “Border” zone. The total distance traveled by mice treated with DMSO or high dose PCIII during the 15-min session (*n* = 4 per group, right panel). (**C**) Relative anxiety was assessed by comparing the time and distance spent between the center and periphery of the open field arena (*n* = 4 mice per group). (**D**) Motor coordination of the mice treated with high dose PCIII or DMSO determined by rotarod test (*n* = 4 mice per group). (**E**) Assessment of potential bradykinesia determined by the pole test (*n* = 4 mice per group). The quantified data are expressed as means ± SEMs. Statistical significance was determined by unpaired two tailed student *t*-test test, NS, nonsignificant.

Dopamine cell loss and Lewy body pathologies progress over several years in humans with PD [1,20]. Therapeutic lead compounds would have to be administered for long periods of time in order to adequately treat PD. Therefore, it is important to ascertain the safety of PCIII treatments in vivo. As compared to the therapeutic dose of PCIII used in Figure 3, we administered 10 times higher dose of PCIII (i.p. 10 mg/kg/day) for seven days to determine whether any toxicity is induced by PCIII treatment in two-month-old C57/BL6 mice. We first measured the body weights of mice treated with either DMSO vehicle or PCIII for seven days. There were no statistical differences in their body weights (Figure 3A). There were also no differences between the spontaneous open field exploration in the two mouse groups (Figure 3B,C). Both groups showed similar total distances of exploration in the open field arena during the 15-min periods (Figure 3B). The tendency to stay in the border of the arena, as an indirect indicator of an anxiety phenotype, was not different between the DMSO and PCIII treated mouse groups (Figure 3C). The groups also showed similar percentages of exploration distance and time in each zone of the “center + periphery” and “border” (Figure 3C).

We next monitored the motor-related behaviors in mice that were treated with high dose PCIII. Their motor coordination was assessed using an accelerated rotarod assay. After brief training on the rotating rod, mice with DMSO or PCIII treatment were placed on the accelerating rotarod. We recorded the latency time when the mice fell from the rotarod, and its speed at that time. Both groups failed to show any statistical difference in motor coordination (Figure 3D). The pole test is commonly used to assess bradykinesia. Both DMSO and PCIII-treated mice displayed similar performance in climbing down the vertical pole (Figure 3E). These behavior experiments indicated the safety of PCIII, with no obvious toxicity on the mice’s motor function.

**Figure 4:**
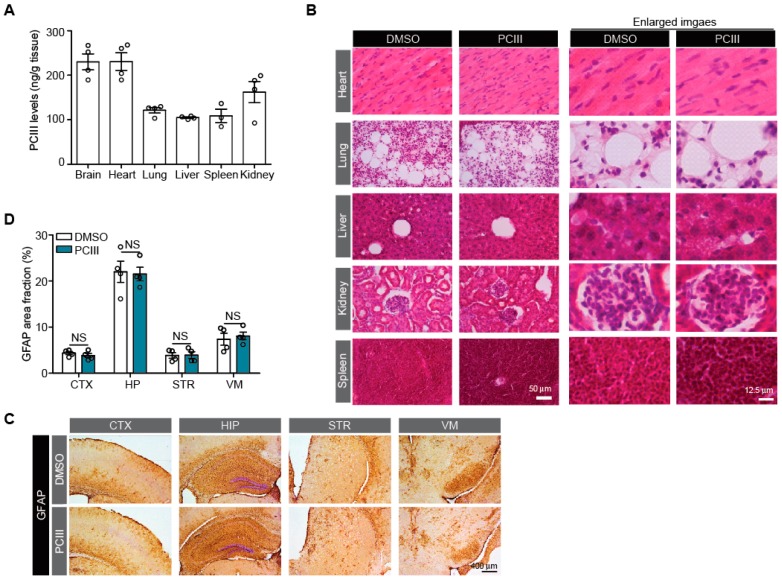
Tissue distribution and potential toxicity of PCIII in vivo. (**A**) Quantification of PCIII levels in each indicated tissue (ng/g tissue weight) determined by UHPLC (*n* = 3 mice for spleen and 4 mice for other tissues) (**B**) Tissue toxicity profiling of PCIII with H&E staining of the indicated tissue sections from mice treated with high dose PCIII (i.p., 10 mg/kg/day) or DMSO. (**C**) Neuropathological assessment of potential neuroinflammation with GFAP immunohistochemistry for the indicated brain sections from mice treated with high dose PCIII (i.p., 10 mg/kg/day) or DMSO. Scale bar = 400 μm (**D**) Quantification of % area fraction occupied by GFAP positive signals in the IHC images of each brain sections of mice treated with high dose PCIII or DMSO as control (*n* = 4 mice per group). The quantified data are expressed as means ± SEMs. Statistical significance was determined by unpaired two tailed student *t*-test test, NS, nonsignificant.

The potential tissue toxicities of high dose PCIII administration (i.p. 10 mg/kg/day, 7 days) were also evaluated in six organs, including the heart, lung, liver, kidney, and spleen. Tissue distribution was first determined using a developed bioanalytical method with ultra high-pressure liquid chromatography (UHPLC) for deproteinated tissue homogenates. PCIII levels in each tissue are shown in Figure 4A. PCIII in all six tissues was readily detectable. Importantly, the therapeutic target tissue, brain, showed 230 ± 36 ng/g brain, suggesting that the PCIII is blood-brain barrier permeable. Also, the PCIII levels were found to be 231 ± 40 ng/g tissue, 121 ± 12 ng/g tissue, 105 ± 3 ng/g tissue, 162 ± 48 ng/g tissue, 109 ± 26 ng/g tissue for heart, lung, liver, kidney, and spleen, respectively (Figure 4A). However, the plasma concentration was observed to be under the lower limit of quantitation (LOQ). With this distribution profile of PCIII in various mouse tissues, histological assessments was performed to evaluate tissue toxicities using hematoxylin and eosin (H&E) staining. H&E staining of these tissue sections from both mice groups revealed that high dose PCIII treatment did not cause any structural alterations in vivo (Figure 4B). The potential PCIII toxicity in brain tissues was determined using GFAP immunohistochemistry to monitor for any ongoing neuroinflammation. GFAP immunostaining was conducted in several brain subregions of both mouse groups, including the cortex, hippocampus, striatum, and ventral midbrain (Figure 4C,D). There was no difference in the GFAP immunohistochemistry in these selected brain subregions of mice that were administered either DMSO or PCIII (Figure 4C,D). These findings indicate that PCIII had no brain toxicity.

## 3. Discussion

Here we report the successful organic synthesis of PCIII racemate. PCIII synthesis was planned using the basic esterification route of trans diol **5** from seselin **4**. Because the preparation routes to seselin **4** from the umbelliferone **2** were already established, we anticipated that large scale synthesis of seselin **4** was possible. Among several known procedures, such as microwave-assisted cyclization, thermal cyclization [9], metal catalyzed cycloisomerization [10], and basic cyclization [11], the thermal cyclization protocol was chosen for our scale-up procedure. This protocol was chosen because of its easily handled purification protocol and high chemical yield. With synthetic seselin **4** in a decagram scale in hand, trans diol **5** was produced after *m*-CPBA mediated epoxidation and the aqueous epoxide opening sequence. We then attempted to perform pivotal selective esterification of the trans diol **5**. Because two alcohols were secondary alcohols, it was difficult to predict which alcohol moiety would react dominantly. In a structural view, the benzylic alcohol is similar to an aromatic ring. Inside the structure, another alcohol is neighbored by a sterically demanding gem-dimethyl group. When tigloylation was carried out under a DMAP-assisted condition, two constitutional isomers **7** and **8** were obtained and purified after careful silica gel chromatography. Unfortunately, the spectral analysis and document search showed that the undesired isomer **7** was a major product. Regarding less reactivity of benzylic alcohol moiety, acetylation/tigloylation of the trans diol **5** sequence was planned. The standard acetylation (Ac_2_O, *i*Pr_2_NEt, DMAP) condition, however, afforded an inseparable mixture of corresponding acetyl isomers along with an unreacted starting material. Because the desired esterification route (acetylation/tigloylation) was unsuccessful, the early procedure (tigloylation/acetylation) route was chosen for large scale synthesis of the desired tigloyl ester **8**. However, this procedure had low isolation yield. Finally, the simple acetylation of the desired tigloyl ester **8** produced PCIII **1** as a racemic mixture. This synthetic PCIII was applied for further biological evaluations, giving similar anti-aggregate activity compared to purified PCIII. It is still a mystery how the synthetic PCIII racemate produced similar therapeutic potential to that of purified PCIII. It would be beneficial to design and validate the organic PCIII synthesis of identical optical stereoisomer structures with purified PCIII. This future study would provide further understanding of the structure and function relationship of PCIII’s stereoisomers and their modes of binding to amyloid protein aggregate. In addition, our successful organic synthesis of PCIII provided reactive intermediates. These intermediate compounds could be applied to compare the structure-activity relationship. They can also be used to synthesize the structural analogs of PCIII to improve its physicochemical properties or therapeutic function.

PCIII was first evaluated for its therapeutic potential in PD mouse models. With only 1 mg/kg administration of PCIII, 6-OHDA induced dopaminergic cell loss and α-synuclein aggregation were diminished substantially. PCIII has been reported to bind protein aggregates, such as α-synuclein aggregate to disentangle them. In doing so, PCIII facilitates proteasomal degradation of protein aggregates [8]. Consistent with previous findings, we showed that in vivo PCIII inhibits pS129-α-Syn-positive Lewy like inclusions in dopamine neurons of PD mouse model. It should be noted that dopaminergic neuroprotection by PCIII administration was not complete compared to the marked inhibition of α-synuclein aggregation. This partial dopaminergic rescue by anti-aggregate compound PCIII might be due to oxidative stress and other signaling alterations caused by 6-OHDA stress. However, this in vivo result is consistent with partial cytoprotection by PCIII in α-synuclein-expressing SH-SY5Y cells challenged with 6-OHDA (Supplementary Figure S1B). This finding suggests the need to apply multiple medications targeting different pathogenic alterations to better control PD. Since PCIII targets α-synuclein aggregates, it is possible to use PCIII in disease-modifying therapies of α-synucleinopathies on various brain subregions. Therefore, future studies are needed to investigate the role of PCIII on other α-synuclein transgenic mice or preformed fibril α-synuclein injection mouse models. An important finding of our study is the in vivo safety validation of PCIII with high dose treatment. Seven days of high dose PCIII administration had no obvious toxic effect on various organs. In addition, PCIII treatment did not cause any inflammatory alterations in diverse brain subregions. At the behavior level, PCIII had no adverse effect on the motor coordination or muscle fine control. Compared to the 1 mg/kg therapeutic dose of PCIII, a 10-times higher dose of PCIII was not found to have any obvious toxicity in vivo. The high therapeutic index of PCIII is advantageous in the treatment of neurodegenerative disorders, which often require repeated medications during disease progression. It is still necessary to test higher doses of PCIII which may give overt toxicity in vivo in order to outline PCIII’s safety index. It would be also critical to evaluate higher dose PCIII treatment in PD mouse models to see if the disease models tolerate the drug with expected therapeutic effect.

Although extensive pharmacokinetic studies of PCIII in the preclinical animals were not performed in this study, we found that PCIII distributed to the brain, the therapeutic target organ, with a concentration of 230 ± 36 ng/g mouse brain (Figure 4A). The extent of tissue distribution of PCIII to brain was higher than those to the liver and lung of mice with 7 days of PCIII treatment. Considering that the blood-brain barrier permeability is an important factor for any compound targeting neurodegenerative diseases, this finding of PCIII distribution suggests that PCIII might be a promising new drug candidate for PD treatment. However, the plasma concentration of PCIII was found to be under lower detection limit of quantitation (i.e., 20 ng/mL). This low plasma concentration of PCIII despite demonstrable distribution of PCIII in other organs remained to be investigated in the future. Moreover, the extensive pharmacokinetic studies including metabolic stability of PCIII may provide instructive translational information prior to clinical evaluation of this drug.

In conclusion, synthetic PCIII racemate holds a great translational value in the treatment of PD pathogenesis including dopaminergic neuron loss and α-synuclein aggregation. It is, however, required to investigate pharmacokinetic properties of PCIII and its target protein binding mode before clinical application.

## 4. Materials and Methods

### 4.1. Chemicals and Antibodies

The PCIII was provided from the National Development Institute of Korean Medicine (NIKOM, Gyeongsan, Korea), as previously described [8]. 6-OHDA, goat serum, and doxycycline were purchased from Sigma. The following primary antibodies were used: mouse antibody to FLAG (M2 clone, 1:5,000, Sigma), mouse antibody to pS129-α-synuclein (#825702, 1:3000, Biolegend, San Diego, CA, USA), rabbit antibody to tyrosine hydroxylase (NB300-109, 1:2000, Novus Biologicals, Centennial, CO, USA), and rabbit antibody to GFAP (#ab7260, 1:1,000, Abcam, Cambridge, MA, USA). The following secondary antibodies were also used: horse radish peroxidase (HRP)-conjugated sheep antibody to mouse IgG (cat# RPN4301, 1:5000, GE Healthcare, Pittsburgh, PA, USA), biotin-conjugated goat antibody to rabbit IgG (cat# BA-1000, 1:1000, Vector Laboratories, Burlingame, CA, USA), Alexa Fluor 488-conjugated goat antibody to rabbit IgG (cat# A11034, 1:1,000; Invitrogen, Carlsbad, CA, USA), Alexa Fluor 568-conjugated donkey antibody to mouse IgG (cat# A11031, 1:1,000; Invitrogen, Carlsbad, CA, USA), and HRP-conjugated mouse antibody to β-actin (cat# A3854, 1:10000, Sigma-Aldrich, St. Louis, MO, USA).

### 4.2. Organic Synthesis of PCIII

The general procedure was performed as follows. For better understanding by readers, we included step-by-step procedure images which were divided and extracted from Figure 1A (full organic synthesis procedure of PCIII) in this method section. Unless noted otherwise, all of the starting materials and reagents were obtained from commercial suppliers and were used without further purification. Tetrahydrofuran and Et_2_O were distilled from sodium benzophenone ketyl. Dichloromethane, triethylamine, acetonitrile, and pyridine were freshly distilled from calcium hydride. All of the solvents used for routine product isolation and chromatography were reagent grade and glass distilled. The reaction flasks were dried at 100 °C. The air and moisture sensitive reactions were performed under argon atmosphere. The chemical shifts are expressed in parts per million (ppm, *δ*) downfield from tetramethylsilane and are referenced to the deuterated solvent (CHCl_3_). The ^1^H-NMR data were reported in the order of chemical shift, multiplicity (s, singlet; d, doublet; t, triplet; q, quartet; m, multiplet and/or multiple resonance), number of protons, and the coupling constant in hertz (Hz).

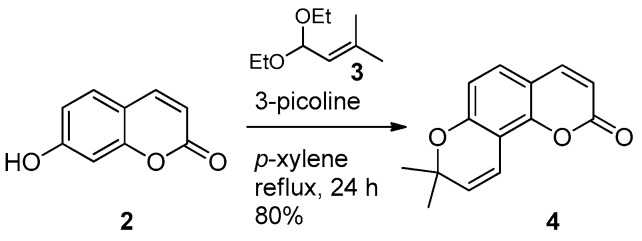



To a solution of umbelliferone **2** (8.6 g, 53 mmol) in *p*-xylene (100 mL), 1,1-diethoxy-3-methyl-2-butene **3** (10 g, 64 mmol) and 3-picoline (1.3 mL, 13 mmol) were added and refluxed. After 24 h, the reaction flask was cooled, diluted with CH_2_Cl_2_, and filtered. The organic residue was evaporated in vacuo and the residue was purified using column chromatography on silica gel (EtOAc: *n*-hexane = 1:5) to present seselin **4** (9.6 g, 80%) as an amorphous solid.

**4**: ^1^H-NMR (CDCl_3_, 300 MHz) δ 7.60 (1H, d, *J* = 9.6 HZ), 7.20 (1H, d, *J* = 8.7 Hz), 6.88 (1H, dd, *J* = 0.6, 8.4 Hz), 6.72 (1H, dd, *J* = 0.6, 8.4 Hz), 6.23 (1H, d, *J* = 9.6 Hz), 5.73 (1H, d, *J* = 9.9 Hz), 1.48 (6H, s); ^13^C-NMR (CDCl_3_, 75 MHz) δ 161.0, 155.3, 150.1, 143.9, 130.7, 127.7, 114.9, 113.5, 112.5 (2C), 109.2, 77.6, 28.1 (2C) HRMS (EI): Calcd. for C_14_H_12_O_3_ ([M]^+^): 228.0786, found: 228.0783.

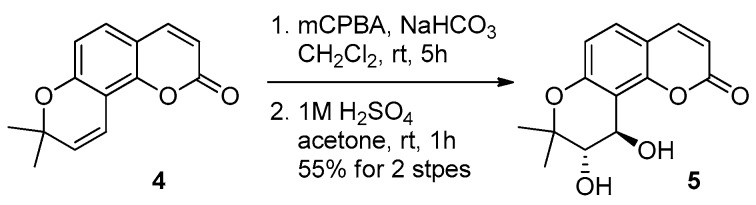



To a solution of seselin **4** (89 mg, 0.39 mmol) in CH_2_Cl_2_ (3 mL), *m*CPBA (77%, 96 mg, 0.43 mmol) and NaHCO_3_ (164 mg, 1.95 mmol) were added at 0 °C. After 5 h of stirring at room temperature, H_2_O was added and extracted with CH_2_Cl_2_ twice. The organic layers were dried over MgSO_4_, filtered, and evaporated in vacuo. The residue was used in the next reaction without further purification. The residue was dissolved in acetone (8 mL). Next, H_2_SO_4_ (1M, 4 mL, 4 mmol) was added to the reaction flask and stirred at room temperature for 1 h. After aq. NaHCO_3_ was added to the reaction mixture, it was transferred to a separatory funnel and extracted with EtOAc twice. The organic layers were dried over MgSO_4_, filtered, and evaporated in vacuo. The residue was purified using column chromatography on silica gel (EtOAc: *n*-hexane = 1:1) to afford diol **5** (57 mg, 55%) as an amorphous solid.

**5**: ^1^H-NMR (CDCl_3_, 300 MHz) δ 7.66 (1H, d, *J* = 12.3 HZ), 7.32 (1H, d, *J* = 8.7 Hz), 6.79 (1H, d, *J* = 8.7 Hz), 6.26 (1H, d, *J* = 9.6 Hz), 5.04 (1H, d, *J* = 6.6 Hz), 3.88 (1H, d, *J* = 6.6 Hz), 3.66 (2H, br), 1.54 (3H, s), 1.32 (3H, s); ^13^C-NMR (CDCl_3_, 75 MHz) δ 161.1, 156.2, 154.2, 144.4, 128.5, 114.8, 112.4, 112.1, 111.7, 79.4, 74.8, 66.5, 25.5, 20.0. HRMS (EI): Calcd. for C_14_H_14_O_5_ ([M]^+^): 262.0841, found: 262.0823.

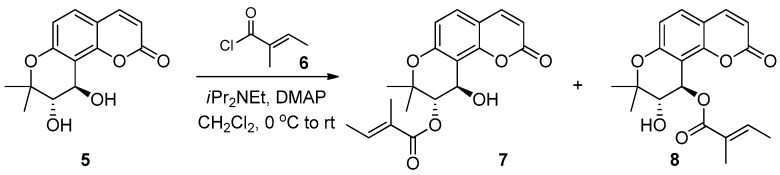



To a solution of diol **5** (52 mg, 0.2 mmol) in CH_2_Cl_2_ (3 mL), *i*Pr_2_NEt (0.070 mL, 0.40 mmol), tigloyl chloride **6** (0.026 mL, 0.24 mmol) and DMAP (2.5 mg, 0.02 mmol) were added at 0 °C. The reaction mixture was warmed to room temperature and stirred for six hours. After adding aq. NH_4_Cl to the reaction mixture, it was transferred to a separatory funnel and extracted with CH_2_Cl_2_ twice. The organic layers were dried over MgSO_4_, filtered, and evaporated in vacuo. The residue was purified by column chromatography on silica gel (EtOAc: *n*-hexane = 1:2) to afford **7** (27 mg, 39%), and **8** (3mg, 4.4%) as amorphous solids.

**7**: ^1^H-NMR (CDCl_3_, 300 MHz) δ 7.62 (1H, d, *J* = 9.6 HZ), 7.30 (1H, d, *J* = 8.1 Hz), 6.78 (1H, d, *J* = 8.4 Hz), 6.76 (1H, ddd, *J* = 12.3, 5.4, 1.8 Hz), 6.21 (1H, d, *J* = 9.3 Hz), 5.20 (1H, d, *J* = 3.3 Hz), 5.04 (1H, d, *J* = 3.3 Hz), 1.76 (3H, s), 1.72 (3H, dd, J = 7.2, 1.0 Hz), 1.47 (3H, s), 1.38 (3H, s); ^13^C-NMR (CDCl_3_, 75 MHz) δ 166.8, 160.8, 155.9, 154.5, 144.0, 139.5, 128.5, 128.0, 114.7, 112.5, 112.4, 110.4, 77.3, 74.0, 62.8, 23.9, 23.8, 14.4, 12.0. HRMS (EI): Calcd. for C_19_H_20_O_6_ ([M]^+^): 344.1260, found: 344.1266.

**8**: ^1^H-NMR (CDCl_3_, 500 MHz) δ 7.58 (1H, d, *J* = 9.5 HZ), 7.33 (1H, d, *J* = 8.0 Hz), 6.84 (1H, d, *J* = 7.0 Hz), 6.79 (1H, d, *J* = 8.5 Hz), 6.21 (1H, d, *J* = 9.5 Hz), 6.08 (1H, s), 3.93 (1H, s), 3.36 (1H, br), 1.82 (3H, s), 1.75 (3H, dd, *J* = 7.0 Hz), 1.46 (3H, s), 1.37 (3H, s); ^13^C-NMR (CDCl_3_, 75 MHz) δ 168.9, 160.1 157.0, 154.3, 143.4, 139.0, 129.1, 128.0, 114.6, 113.0, 112.5, 107.4, 79.1, 73.2, 68.5, 24.7, 21.3, 14.5, 12.1. HRMS (EI): Calcd. for C_19_H_20_O_6_ ([M]^+^): 344.1260, found: 344.1256.

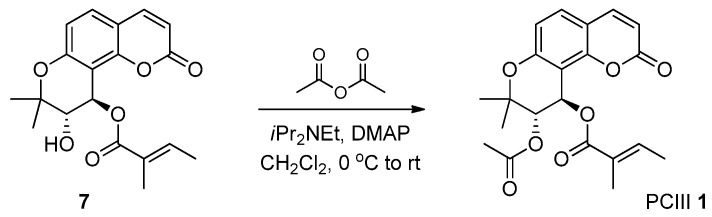



To a solution of **7** (4 mg, 0.012 mmol) in CH_2_Cl_2_ (2 mL), *i*Pr_2_NEt (6 drops, excess), acetic anhydride (4 drops, excess), and DMAP (1 mg, 0.08 mmol) were added at 0 °C. The reaction mixture was warmed to room temperature and stirred for six hours. After adding aq. NH_4_Cl to the reaction mixture, it was transferred to a separatory funnel and extracted with CH_2_Cl_2_ twice. The organic layers were dried over MgSO_4_, filtered, and evaporated in vacuo. The residue was purified by column chromatography on silica gel (EtOAc: *n*-hexane = 1:2) to afford PCIII **1** (4 mg, 89%) as an amorphous solid.

PCIII **1**: ^1^H-NMR (CDCl_3_, 500 MHz) δ 7.57 (1H, d, *J* = 9.5 HZ), 7.35 (1H, d, *J* = 9.0 Hz), 6.81 (1H, d, *J* = 8.5 Hz), 6.78 (1H, dq, *J* = 7.5, 1.0 Hz), 6.20 (1H, d, *J* = 8.5 Hz), 6.20 (1H, d, *J* = 3.0 Hz), 5.30 (1H, d, *J* = 3.5 Hz), 2.06(3H, s), 1.82 (3H, t, *J* = 1.0 Hz)), 1.73 (3H, dd, *J* = 7.0, 1.0 Hz), 1.44 (3H, s), 1.36 (3H, s); ^13^C-NMR (CDCl_3_, 125 MHz) δ 169.3, 166.3, 159.8, 156.5, 154.3, 143.1, 138.0, 129.1, 128.1, 114.4, 113.3, 112.4, 106.6, 77.0, 71.3, 63.3, 23.8, 23.7, 20.7, 14.4, 12.1. HRMS (EI): Calcd. for C_21_H_22_O_7_ ([M]^+^): 386.1366, found: 386.1348.

### 4.3. Cell Culture and Transfection

We maintained human neuroblastoma SH-SY5Y cells (ATCC, Manassas, VA) in DMEM containing 10% FBS (*v/v*) and antibiotics (penicillin-streptomycin 100 U/mL, ThermoFisher Scientific). The cell culture incubator was set with a humidified atmosphere consisting of 5% CO2/95% air at 37 °C. To transiently transfect the indicated plasmids (pCMV-tTA, TetP-β23, pTet-dual2, and HA-α-synuclein [8]) to SH-SY5Y cells, the X-tremeGENE HP transfection reagents (Roche) were used following the manufacturer’s protocol.

### 4.4. Fluorescence Imaging

The SH-SY5Y were plated onto poly-*D*-lysine-coated coverslips at a density of 10,000 cells/cm^2^. Following the experimental procedures (Figure 1B), we fixed the cells with 4% paraformaldehyde in PBS and blocked in a solution containing 5% normal goat serum (Sigma-Aldrich, St. Louis, MO, USA) and 0.1% Triton X-100 (Sigma) for 1 h at room temperature. The fixed cells were then incubated overnight with their corresponding primary antibodies against FLAG-tagged β23 at 4 °C. After three brief washes with PBS containing 0.1% Triton X-100, we incubated the samples for one hour with fluorescence-conjugated secondary antibodies (1:500; Invitrogen, Carlsbad, CA, USA) at room temperature. Coverslips were then mounted with 4’,6-diamidino-2-phenylindole (DAPI) for nuclear counter staining. The fluorescent images acquisition was done by using a fluorescence microscope (Axiovert, 200 M; Carl Zeiss, Germany).

### 4.5. Western Blotting

For the preparation of total protein lysates, we added lysis buffer (1% Nonidet P40 in phosphate-buffered saline (PBS), pH 7.4) that was supplemented with protease/phosphatase inhibitors to the SH-SY5Y cells after a brief wash with ice cold PBS. After three freeze and thaw cycles in dry ice and iced water for complete lysis of cells, respectively, the samples were centrifuged at 14,000× *g* for 30 min. The supernatants were mixed with the same volume of 2X Laemmli buffer (Bio-Rad, Hercules, CA, USA) supplemented with β-mercaptoethanol (Sigma-Aldrich, St. Louis, MO, USA), and boiled for five minutes. The proteins were separated by SDS-PAGE and transferred to nitrocellulose membranes for immunoblotting. The blotted nitrocellulose membranes were stained with Ponceau (Sigma) to verify the uniform protein transfer. The nitrocellulose membrane was incubated with the designated antibodies, and immunoreactive bands were visualized via chemiluminescence (Pierce). Next, we performed densitometric analyses of the bands using ImageJ (NIH, http://rsb.info.nih.gov/ij/).

### 4.6. Cell Viability Assay

We plated SH-SY5Y cells on 6-well plates at a density of 0.5 × 10^6^ cells per well. Following transient transfection with the indicated constructs and subsequent treatment (Figure 1B, and Supplementary Figure S1), the cells were harvested as single cell suspensions by trypsinization. After two PBS washes, the cells were resuspended in serum-free DMEM. The resuspended cells were mixed with an equal volume of 0.4% trypan blue (*w/v*) and incubated for 2 min at room temperature. Live and dead cells were counted using a Countess II Automated Cell Counter (Life Technologies, Bothell, WA, USA) for cell viability measurement.

### 4.7. Animal Experiments

All of the animal experiments were approved by the Ethical Committee of Sungkyunkwan University (Approval #: SKKUIACUC2019-06-15-1; Approval date: 2019-07-02) and were conducted in accordance with all applicable international guidelines. Male C57BL/6N mice (2 months old) was obtained from Orient Bio (Suwon, Korea). The animals were housed in air-controlled rooms with a 12-h dark/light cycle. Mice and rats were provided ad libitum access to diet and water. All efforts were made to minimize the animals’ suffering and the number of animals used in experiments. Synthetic PCIII racemate was administered to mice intraperitoneally (i.p.). The i.p. PCIII administration (1 mg/kg/day for 6-OHDA PD mouse models; 10 mg/kg/day for tissue distribution and toxicity experiments) began on day 0 and was continued for 7 days. Intrastriatal injection of 6-OHDA was performed on day 0. The mouse brains were prepared for analysis as described below. To determine the tissue distribution and toxicity of PCIII in vivo, the brains and other biological tissue samples were collected at 30 min after the last administration of PCIII.

### 4.8. UHPLC Bioanalytical Method of PCIII

For the determination of levels of tissue concentration for PCIII, the mouse tissues including brain were homogenized by adding two-fold volume of PBS, and then the tissue homogenates were deproteinized by adding two-fold volume of acetonitrile. After centrifugation at 15,000 rpm for 15 min at 4 °C, the supernatant was taken for the next analysis. A UHPLC-DAD method for PCIII was established using an UHPLC system comprising of the Agilent 1290 Infinity II UHPLC system (Agilent Technologies, Santa Clara, CA, USA), equipped with an auto-sampler (G7167B), a flexible pump (G7104A), a Multicolumn Thermostat (MCT) (G7116B), and a DAD detector (G7117A). The separation between PCIII and endogenous substances derived from plasma was accomplished using a Capcellpak^TM^ C18 5 µm column (UG120; 250 × 1.5 mm, Shiseido Fine Chemical Co., Tokyo, Japan). A gradient condition was applied with a mixture of 0.1% phosphoric acid and acetonitrile at a flow rate 0.2 mL/min. The percentage of mobile phase (0.1% phosphoric acid and acetonitrile) was as follows; from initial to 1 min, 50:50; from 1 to 3 min, 15:85; from 3 to 7 min, 15:85; from 7 to 9 min, 50:50; from 9 to 16 min, 50:50. The DAD detector was set to 321 nm and injection volume was 10 µL. Calibration curve was set up in a range covering from 20 to 1000 ng/mL for PCIII. The linearity, precision, and accuracy for PCIII were observed within the acceptable ranges. The tissue concentration level was expressed as a unit of PCIII amount (ng)/g tissue, as described previously [21].

### 4.9. Intrastriatal Injection of 6-OHDA

Intrastriatal injeciton of 6-OHDA was performed as previously described [22] with some modifications. For stereotaxic injection of 6-hydroxy dopamine (6-OHDA, 8 µg), 2-month-old C57/BL6N mice treated either with PCIII for 7 days or DMSO as a control were anesthetized with alfaxalone (100 mg/kg). An injection cannula (26.5 gauge) was applied stereotaxically into the striatum (anteroposterior, 0.5 mm from bregma; mediolateral, 2.0 mm; dorsoventral, 3.0 mm) and unilaterally applied into the right hemisphere. The infusion rate was 0.2 µl/min with total injection volume of 2 µl of 6-OHDA (4 µg/µl in sterile PBS) into each mouse. After the 6-OHDA injection was completed, the cannula was maintained in the striatum for an additional 5 min to facilitate complete absorption of the 6-OHDA to brain tissues. The cannula was then slowly removed from the mouse brain. The skin over the skull was closed by suturing. We closely monitored wound healing and recovery of mice following the surgery. For the stereological analysis, the animals were perfused at 7 days after intrastriatal 6-OHDA injection and fixed intracardially with ice-cold PBS followed by 4% paraformaldehyde. The tissue was post-fixed with 4% paraformaldehyde overnight. The fixed mouse brains were cryoprotected in 30% sucrose (*w/v*) in PBS and subsequently processed for immunohistochemistry.

### 4.10. TH Stereological Cell Counting and Immunohistochemistry

TH setereological assessment was carried out as previously described [22]. Briefly, coronal sections (thickness of 40 um) of the fixed and cryoprotected brains were cut, including the substantia nigra. Every fourth section was used for analysis. To analyze the tyrosine hydroxylase (TH) expression, the sections were incubated with a 1:1000 dilution of rabbit polyclonal anti-TH (Novus) antibody followed by sequential incubations with biotinylated goat anti-rabbit IgG and streptavidin-conjugated horseradish peroxidase (HRP) using a Vectastain ABC kit (Vector Laboratories, Burlingame, CA, USA) according to the manufacturer’s instructions. To visualize the TH-positive cells, 3,3-diaminobenzidine (DAB, cat# D4293, Sigma-Aldrich, St. Louis, MO, USA) was used as an HRP substrate. The total number of TH-positive neurons in the substantia nigra pars compacta was determined using the Optical Fractionator probe in Stereo Investigator software (MicroBrightfield, Williston, VT, USA). All of the stereological counting was performed in a blinded manner to the mouse treatments. For immunofluorescence labeling, coronal ventral midbrain sections were blocked in PBS containing 4% normal goat serum and 0.2% Triton-X for 1 h at room temperature. The brain sections were incubated with the indicated antibodies overnight at 4 °C, followed by a brief washing and subsequent incubation in PBS containing fluorescent dye labeled secondary antibodies. The fluorescent images were obtained using a fluorescence microscope (Axiovert, 200 M; Carl Zeiss, Germany) for image acquisition.

### 4.11. Behavior Tests

A rectangular wood box (40 cm × 40 cm × 40 cm) divided into 64 (8 × 8) identical regions (5 cm × 5 cm) was used as an open arena for the open field behavior test. The field was subdivided into border, peripheral, and central sectors automatically by a video tracking system (Smart v3.0 software). The central region included 4 central squares (2 × 2), the peripheral region included 12 squares surrounding the center region, and the border regions made up the remaining squares. The mouse was placed at the center of an open field and allowed to explore for 15 min under dim light. We cleaned the open field arena with diluted 70% ethanol between each trial. A video tracking system (Smart v3.0 software) was used to record the distance traveled as a measure of locomotor activity. The time spent in and entries into the center were measured as an anxiolytic indicator. The mice were trained 2 days before the rotarod test. On day 3, mice were placed on an accelerating rotarod cylinder, and the latency time at which they fell off the machine was measured. The speed of the rotarod was slowly increased from 4 to 40 rpm within 5 min. A trial ended if the animal fell from the rotarod. The test data are presented as means of latency time (3 trials). For the pole test, the animals were allowed to acclimate to the behavioral procedure cage for at least 30 min. The pole is made up of 58 cm of metal rod with 10 mm diameter with bandage gauze wrapped around the pole. Briefly, the pole was placed inside the acclimatized cage, and the mice were placed on the top portion of the pole facing head-down. The total time that it took the animals to reach the base of the pole was recorded. The mice were evaluated in three sessions, and the average time was recorded.

### 4.12. Tissue Pathological Staining

4% PFA fixed and 30% sucrose embedded tissue sections were sliced at 40 um thickness for pathological assessment. The tissue sections mounted onto glass slides (Fisher Scientific) were processed for hematoxylin and eosin (H&E) staining. After dehydration step, the sections were stained with hematoxylin (Merck, Darmstadt, Germany) for 3 min and eosin (Merck, Darmstadt, Germany) for 1 min, respectively. The sections were destained by rinsing with gradually increasing the concentrations of ethanol/water solutions and then mounted with DPX mounting medium (Sigma-Aldrich) after the fixation in xylene. For the neuropathological assessments, GFAP immunohistochemistry was performed by the procedures described in the method Section 4.10. Bright field images were obtained using a microscope (Axiovert, 200 M; Carl Zeiss, Germany).

### 4.13. Statistics

The quantitative data are presented as means ± SEMs. Power analysis was done by using G*Power 3.1 software to estimate the approximate sample sizes for tyrosine hydroxylase stereological counting. On the basis of the mean difference from our preliminary experiments and related previous report [22], a total sample size of four mice was calculated to potentially obtain significant differences (effect size f = 22.42 for 45% mean difference; α = 0.05). The statistical significance was assessed using an analysis of variance (ANOVA) test with Tukey’s HSD post hoc analysis (comparisons of more than three groups). Differences with a *p* value < 0.05 were considered statistically significant. We used GraphPad Prism software to prepare all of the plots and statistical analyses.

## Supplementary Materials

Supplementary Materials can be found at https://www.mdpi.com/1422-0067/20/21/5481/s1.

## Abbreviations

PD	Parkinson’s disease
Ac_2_O	Acetic anhydride
*m*-CPBA	*meta*-Chloroperoxybenzoic acid
6-OHDA	6-hydroxydopamine
