# Supplementary material for: Therapeutic Evaluation of Synthetic Peucedanocoumarin III in an Animal Model of Parkinson’s Disease
Source: Int J Mol Sci. 2019 Nov 4;20(21):5481. doi: 10.3390/ijms20215481 (PMC6862101; doi:10.3390/ijms20215481)
Supplement: Supplementary file 1 [file ijms-20-05481-s001.pdf]

SUPPLEMENTAL INFORMATION

Therapeutic evaluation of synthetic peucedanocoumarin III in an animal model of Parkinson's disease

Sangwoo Ham, Heejeong Kim, Jin-Ha Yoon, Hyojung Kim, Bo Reum Song, Jeong-Yun Choi, Yun-Song Lee, Seung-Mann Paek, Han-Joo Maeng, and Yunjong Lee

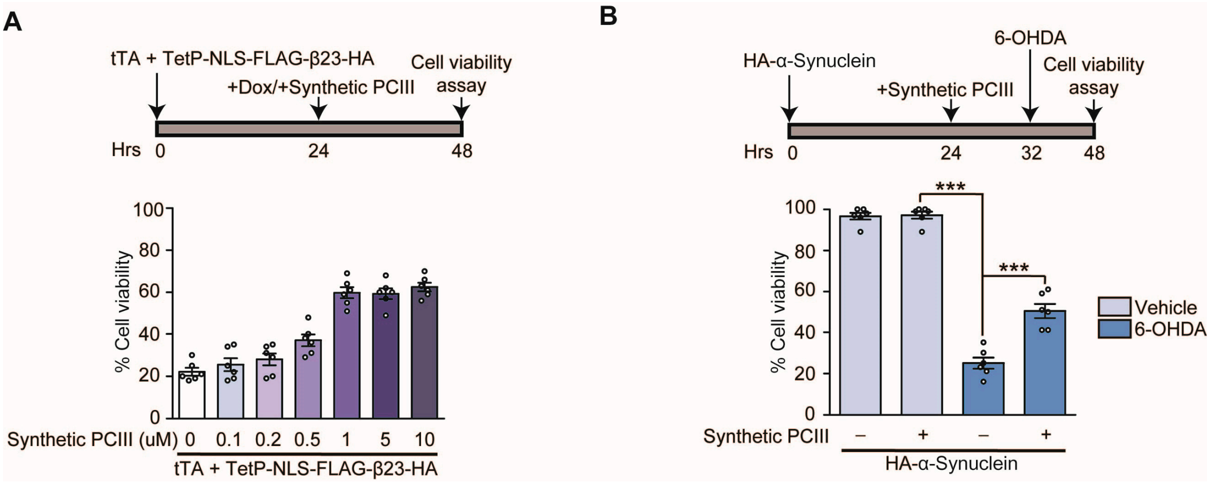

Supplementary figure 1. PCIII-mediated cytoprotection against amyloid aggregate and oxidative stress

(A) Trypan blue exclusion cell viability assessment demonstrating cytoprotection by increasing concentrations (0.1, 0.2, 0.5, 1, 5, 10 μM) of synthetic PCIII against nuclear β23 expression in SH-SY5Y cells (*n* = 6). Experimental schedule is illustrated in the top panel.

(B) Trypan blue exclusion cell viability assessment demonstrating cytoprotection by synthetic PCIII (1 μM) against 6-OHDA (70 μM, 16 h)-induced oxidative stress in SH-SY5Y cells transfected with HA-α-synuclein (*n* = 6). Experimental schedule is illustrated in the top panel.

The quantified data are expressed as means ± SEMs. Statistical significance was determined by the ANOVA test with Tukey post-hoc analysis, ***$p < 0.001$.
